# The Utility of Endoscopic Ultrasound Fine Needle Aspiration in Pancreatic Cystic Lesions Diagnosis

**DOI:** 10.3390/diagnostics10080507

**Published:** 2020-07-22

**Authors:** Tawfik Khoury, Anas Kadah, Amir Mari, Bahir Sirhan, Mahmud Mahamid, Wisam Sbeit

**Affiliations:** 1 Department of Gastroenterology, Galilee Medical Center, Nahariya 22100, Israel; anas18kadah@msn.com (A.K.); bahir.sirhan@gmail.com (B.S.); wisams@gmc.gov.il (W.S.); 2 Faculty of Medicine in the Galilee, Bar-Ilan University, Safed 1311502, Israel; 3 Gastroenterology and Endoscopy United, The Nazareth Hospital, EMMS, Nazareth 16100, Israel; amir.mari@hotmail.com; 4 Faculty of medicine, Bar-Ilan University, Safed 1311502, Israel; 5 Department of Gastroenterology, Shaare Zedek Medical Center, Jerusalem 9103102, Israel; mahmudmahamid@yahoo.com

**Keywords:** cysts, pancreas, FNA, yield, EUS

## Abstract

The yield of biochemical analysis of pancreatic cysts fluid obtained via fine needle aspiration (FNA) is limited. We aimed to assess whether biochemical cyst analysis correlates with the endoscopic ultra-sonographic (EUS) diagnosis. A retrospective study including patients who underwent EUS-FNA was performed. Agreement level between EUS diagnosis and biochemical analysis was reported. One-hundred-and-eleven patients were included. For cyst CEA level, 42.4% of patients with endoscopic diagnosis of pancreatic mucinous cystic neoplasm (intraductal papillary mucinous neoplasm (IPMN) and mucinous cystic neoplasms (MCN)) had CEA level >192 ng/mL vs. 15.8% of patients who had another endoscopic diagnosis (chi square = 0.03) with poor agreement level (Kappa = 0.130). For the serous cystadenoma (SCA), the levels of amylase and CEA were defined as <250 unit/L and <5 ng/mL, respectively. Eight patients (57.1%) had amylase of <250 unit/L, while 42.9% had >250 unit/L (chi square = 0.007). The agreement level between EUS diagnosis of SCA and amylase level was poor (Kappa = 0.231). For cyst CEA level, 71.4% had CEA level <5 ng/mL vs. 28.6% who had CEA >5 ng/mL (chi square < 0.001) with fair agreement level (Kappa = 0.495). EUS-FNA for pancreatic cystic lesions poorly correlated with the EUS diagnosis. FNA should be considered in the setting of EUS worrisome findings.

## 1. Introduction

Cystic pancreatic lesions are increasingly diagnosed due to the widespread availability of abdominal imaging. It is estimated that they are discovered incidentally in about 2.5% of these imaging tests and their frequency increases with age to about 10% in those older than 70 of age [1,2]. They could be pseudocysts (PC), benign or malignant cysts, and thus when encountered, they represent a diagnostic and therapeutic challenge [3]. Pancreatic cystic lesions are mainly classified into intraductal papillary mucinous neoplasm (IPMN, 38%), mucinous cystic neoplasms (MCN, 23%), serous cystic adenoma (SCA, 16%), and cystic neuroendocrine neoplasms (7%) [4]. The diagnosis of these pancreatic cysts is mostly made by imaging studies, including computed tomography (CT), magnetic resonance imaging (MRI) and endoscopic ultrasound (EUS). The proximity of the EUS transducer to the pancreas makes it a potentially ideal modality for inspecting the pancreas, including cyst size, number, localization, locularity, calcifications, cystic wall thickness, intra-nodules, pancreatic duct width, and connection between the cysts and the pancreatic ducts. The reported diagnostic accuracy of EUS morphology is wide-ranging from 51% to 90% [5]. However, the utility of EUS-fine needle aspiration (FNA) in the diagnosis of pancreatic cystic lesions is still unknown [6]. It has been reported that the addition of EUS-FNA to CT and MRI increased the overall accuracy for diagnosing cystic pancreatic neoplasms by 36% and 54%, respectively [7]. However, there is still a controversy about the role of EUS-FNA in diagnosing cystic pancreatic neoplasms due to the low yield of cyst fluid cytological and biochemical analysis. One previous meta-analysis reached the conclusion that EUS-FNA has low sensitivity but high specificity [8]. Given this uncertainty about the diagnostic yield of EUS-FNA in pancreatic cysts, we aimed to investigate the yield of EUS-FNA in pancreatic cystic lesions and to assess whether the cyst fluid analysis correlates with the morphologic diagnosis as demonstrated by endoscopic ultra-sonography.

## 2. Methods

We performed a cross-sectional, retrospective study, using the databases of Galilee Medical Center. All patients who underwent EUS-FNA for investigating a pancreatic cystic lesion between 2011–2019 were included in the study. Inclusion criteria were patients 18 years of age or older who were referred to EUS for investigation of pancreatic cystic lesions and who underwent FNA. Exclusion criteria included solid pancreatic lesions and contraindication for FNA such as intervening vessels and coagulopathy. Extracted data included demographic variables (age, gender), cyst characteristics including maximal cyst size, wall thickening, calcifications, cyst nodule, and Wirsung width, in addition to cyst fluid analysis results including *Carcinoembryonic antigen* (CEA), amylase and cytology. All procedures were carried out via linear echoendoscope (Pentax-Japan) model 3870 and performed by a single gastroenterologist with more than 15 years’ experience in the field of endoscopic ultrasound. Patients were placed in the left lateral decubitus position and were sedated with intravenous midazolam and propofol, according to the decision of the endoscopist. Following identification of a cyst, EUS-FNA was performed after ensuring the absence of intervening vessels by Doppler ultrasound. All FNAs were obtained via 22 Gauge needle (COOK MEDICAL, echo tip ultra, Bloomington, Indiana 47402-1608 USA). The study was carried out following the rules of the Declaration of Helsinki of 1975. The study was approved by the local institutional ethics committee. Written informed consent was waived due to the retrospective, non-interventional study design. 

### 2.1. Morphologic Sonographic Characterization of Pancreatic Cyst

Four types of pancreatic cysts were reported in our study. The morphologic sonographic diagnosis of the pancreatic cysts in our study was made according to the following characteristics: (1) IPMNs-main-duct IPMNs are characterized by pancreatic duct dilatation in EUS without obstructing lesion or stone while branch-duct type is characterized by cystic dilation of branch ducts connected to the main pancreatic duct. Mixed type represents a combination of both types [9]. (2) MCN-female dominance typically consists of multiple macrocystic locules but may be unilocular, most commonly located in the body or tail of pancreas and characterized by cystic lesion not connected to the pancreatic ducts. It contains pathognomonic peripheral wall calcification in about 15% of cases [9,10]. (3) SCA-focal lesions that may be located anywhere in the pancreas, usually consisting of multiple small cysts separated by septa, resembling a honeycomb with pathognomonic central calcification in up to 20% of cases [11]; and (4) PC—mostly extra-pancreatic cyst with thin muddy-brown debris occurring in a patient with history of moderate to severe pancreatitis or abdominal trauma [12].

### 2.2. Chemical Diagnosis

The cyst fluid was analyzed for amylase and CEA level. Unfortunately, reported sensitivities and specificities of chemical analyses have broad ranges, making interpretation difficult [13,14]. A prospective, multicenter study of 112 pancreatic cysts diagnosed by surgical resection or biopsy, found an optimal CEA cutoff of 192 ng/mL for differentiating mucinous from non-mucinous cysts, providing a sensitivity of 75%, a specificity of 84% [14] and CEA level of <5 ng/mL for serous cystadenoma. Accordingly, the American Society of Gastrointestinal Endoscopy (ASGE) guidelines recommend a cut-off level of CEA of >192 ng/mL for pancreatic mucinous cysts and CEA <5 ng/mL for serous cysts [12]. It does not recommend a cut-off level for cyst amylase. However, a previous study by Van Der Waaji et al. reported a cut-off value for amylase in SCA of <250 unit/L [15]. Similarly, the World Gastroenterology Guidelines report a CEA level >192 ng/mL for mucinous cysts IPMN and MCN (sensitivity 73%, specificity 84%) and <5 ng/mL for serous cysts (sensitivity 0.1 and specificity 0.86) (WGO Global Guideline Pancreatic Cystic Lesions 2019). For PC, The European Study Group on Cystic Tumors of the Pancreas guidelines reported that low level amylase may exclude pancreatic pseudocysts (amylase <250 U/L; sensitivity 0.44, specificity 0.98) [16]. Therefore, in our study, the agreement between the morphologic sonographic diagnosis and the FNA cyst fluid analysis results diagnosis was set at CEA >192 ng/mL for mucinous cysts. CEA <5 ng/mL and amylase <250 unit/L diagnosis for SCA and amylase <250 unit/L for PC. CEA was assessed with the ARCHITECT CEA assay, which is a two-step immunoassay using chemiluminescent microparticle immunoassay (CMIA) technology with flexible assay protocols, referred to as Chemiflex. In the first step, sample and anti-CEA-coated paramagnetic microparticles are combined. CEA present in the sample binds to the anti-CEA coated microparticles. After washing, anti-CEA acridinium-labeled conjugate is added in the second step. Pre-Trigger and Trigger Solutions are then added to the reaction mixture; the resulting chemiluminescent reaction is measured as relative light units (RLUs). The amylase was assessed by the ARCHITECT amylase assay by hydrolyzing the 2-chloro-4-nitrophenyl-α-D-maltotrioside (CNPG3) by α-Amylase to release 2-chloro-4-nitrophenol (CPNP) and form 2-chloro-4-nitrophenyl-α-D-maltoside (CNPG2), maltotriose and glucose. The rate of formation of the 2-chloro-4-nitrophenol was detected spectrophotometrically at 404 nm, which gives a direct measurement of α-amylase activity in the sample. 

### 2.3. Cytological Diagnosis

The specimens of all patients were sent for cytological examination. The cytopathology was assessed by a single cytopathologist with more than 20 years’ experience in the field of pancreatic diseases. The cytopathologist reported the adequacy of the specimen and, when possible, the final pathological diagnosis. Any specimen with inadequate reporting was considered non-informative for the purposes of our study.

### 2.4. Statistical Analysis

Chi-square and Fisher’s exact tests were used to analyze the association between two categorical variables, which was presented as percentages, while two-sample *t*-test was used to compare continuous variables. Statistically significant *P* values are set at *p* < 0.05. The “agreement” between diagnosis according to cyst fluid analysis results and the morphologic sonographic diagnosis that may occur over and above that, which would have been expected by chance, was assessed by the “kappa” statistic. A kappa value of ≤0.20 is considered as “poor” agreement, 0.21 to 0.40 as “fair,” 0.41 to 0.60 as “moderate,” 0.61 to 0.80 as “good”, and ≥0.81 as an excellent agreement. The cut-off points for cyst CEA and amylase levels were determined using receiver operating characteristics (ROC) analysis with the Youden index (J) reported. We determined the diagnostic accuracy of the cut-off point generated using sensitivity and specificity. The analysis was conducted using IBM SPSS statistical software, Armonk, New York 10504-1722, United States (version 9.0). 

## 3. Results

### 3.1. Demographics, Chemical and Morphologic Sonographic Characteristics

One-hundred-and-eleven (111) patients who underwent EUS-FNA for pancreatic cystic lesions were included in the study. According to the endoscopic diagnostic criteria mentioned above, we had 81 patients (73%) diagnosed with IPMN (group A), 14 patients (12.6%) diagnosed with SCA (group B), 11 patients (9.9%) diagnosed with MCN (group C), and 5 patients (4.5%) diagnosed with PC (group D). The average age (years) was 72 ± 11.1, 62.6 ± 14.7, 66.4 ± 11.2 and 56.2 ± 16.6 for groups A, B, C, and D, respectively. Female gender was more common in groups A, B and C (47 patients (58%), nine patients (64.3%) and seven patients (63.6%)), respectively, while all patients in group D were males. The mean cyst amylase was significantly higher in groups A, C and D (49,288 unit/L, 10,981.9 unit/L and 19,452 unit/L, respectively), than in group B (805.7 unit/L). Similarly, the cyst CEA level was higher in groups A and C as compared to groups B and D (978.2 ng/mL and 219.3 ng/mL vs. 116.5 ng/mL and 164.7 ng/mL). Table 1 demonstrates the demographics and endoscopic and chemical tests findings.

### 3.2. Agreement between Endoscopic Ultrasonographic Diagnosis of Mucinous Cysts (IPMN and MCN) with Cyst CEA Level

Cyst CEA level for mucinous cysts as reported in the literature that support IPMN and MCN diagnosis is >192 ng/mL. For cyst CEA level, 42.4% of patients with morphologic sonographic diagnosis of pancreatic mucinous cystic neoplasm (IPMN and MCN) had CEA level >192 ng/mL compared to 15.8% of patients who had another morphologic sonographic diagnosis, but with CEA >192 ng/mL (chi square = 0.03). The agreement level between morphologic sonographic diagnosis of IPMN and CEA >192 ng/mL was poor (Kappa coefficient correlation = 0.130). For morphologic sonographic diagnosis of IPMN alone, 36 patients (44.4%) had CEA level >192 ng/mL compared to 6 patients (20%) with morphologic sonographic diagnosis (chi square = 0.018) with accuracy of 54%. However, the agreement level was poor (Kappa coefficient correlation = 0.173). Similarly, for morphologic sonographic diagnosis of MCN, 3 patients (27.3%) had CEA level >192 ng/mL compared to 39 patients (39%) who had another morphologic sonographic diagnosis (chi square = 0.44), with poor agreement level (Kappa coefficient correlation = 0.130). (Table 2)

### 3.3. Correlation between Morphologic Ultrasonographic Diagnosis of SCA and Chemical Analysis (Amylase and CEA)

Among the SCA patients (group B), we defined a level of both amylase and CEA as reported in the literature that supports SCA diagnosis (amylase level <250 unit/L and CEA level <5 ng/mL). Among patients with SCA, eight patients (57.1%) had cyst amylase of <250 unit/L, while 42.9% had amylase level >250 unit/L with accuracy of amylase level <250 unit/L for SCA of 74.8% (chi square = 0.007). However, the agreement level between EUS diagnosis of SCA and amylase level was poor (Kappa coefficient correlation = 0.231). For cyst CEA level, 71.4% of patients with morphologic sonographic diagnosis of SCA had CEA level <5 ng/mL compared to 28.6% of patients who had CEA >5 ng/mL (chi square < 0.001) with accuracy of 86.5%. The agreement level between EUS diagnosis of SCA and CEA level <5 ng/mL was fair (Kappa coefficient correlation = 0.495). When assessing SCA diagnosis with either positive test, we found that 11 patients (78.6%) diagnosed with SCA had one test positive compared to 3 patients (21.4%) who had both tests negative (chi square < 0.001) with accuracy of 71.2%. However, the level of agreement between SCA and either test was poor (Kappa coefficient correlation = 0.271). Combining the two tests, we found that among patients with SCA, 50% had both tests positive compared to 4.1% with another diagnosis (chi square < 0.001), with accuracy rate of 90.1% and with fair agreement level (Kappa coefficient correlation = 0.505). (Table 3)

### 3.4. Subgroup Analysis of Positive Cytology Group

**Figure 1:**
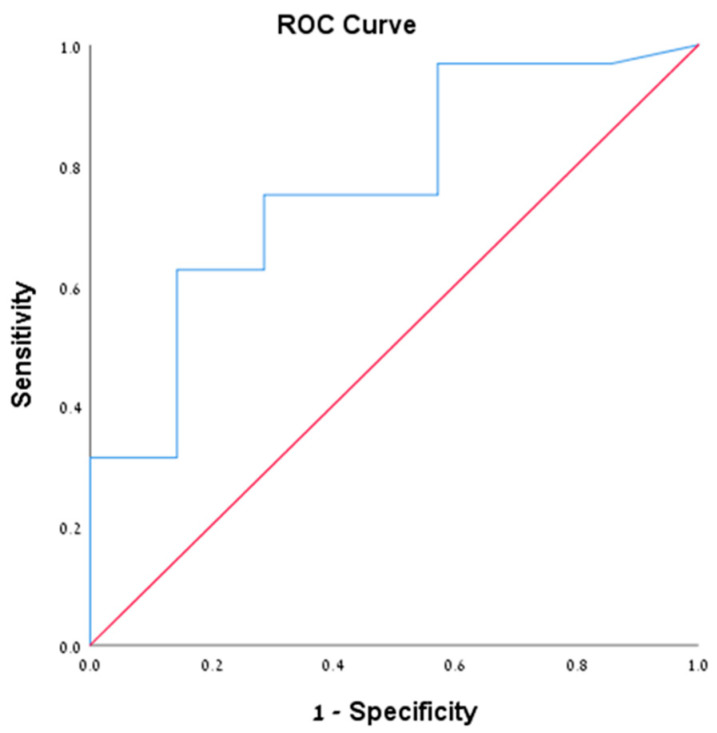
Demonstrating the ROC of CEA level of 25.5 ng/mL in the cytologically positive group.

**Figure 2:**
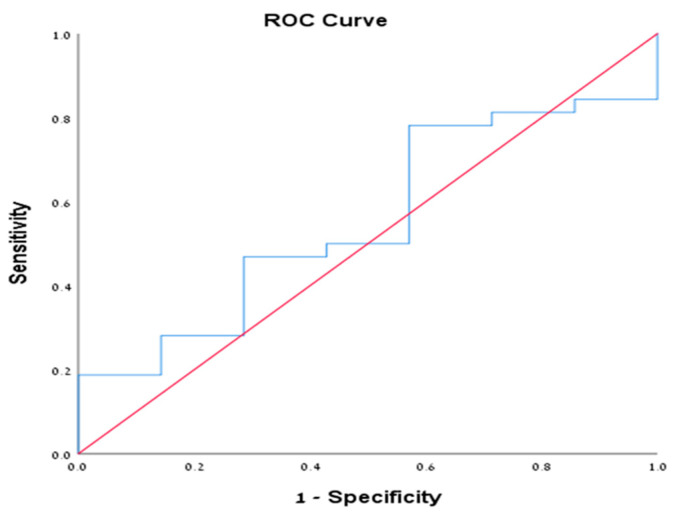
Demonstrating the ROC of cyst amylase in the cytologically positive group.

We performed sub-group analysis on patients with positive cytology (39 patients from all cohorts, 35.1%). Of them, 32 patients (82%) were diagnosed with mucinous cystic neoplasms (IPMN and MCN). The mean level of cyst amylase and CEA were 50270.6 and 1068.9, respectively. Among them, 13 patients (40.6%) had CEA level >192 ng/mL compared to only 1 patient (14.3%) who had another endoscopic diagnosis (chi square = 0.188) (Table 4). Furthermore, we performed ROC analysis for both amylase and CEA in this subset of patients given the morphologic sonographic diagnosis of mucinous cysts is confirmed by cytological examination. The ROC of CEA was 0.766 (95% CI 0.575–0.956) (Figure 1), with CEA value according to the Youden index of 25.5 ng/mL or more that was associated with sensitivity of 0.750 and specificity of 0.714. While for cyst amylase level, the ROC was 0.554 (95% CI 0.335–0.772) (Figure 2), with amylase value according to the Youden index of 181 unit/L or more that was associated with sensitivity of 0.781 and specificity of 0.429, suggesting that CEA is more specific than amylase for mucinous cysts. Notably, all patients who had positive cytology for mucinous cysts had accurate morphological diagnosis, suggesting the reliability of the EUS morphological criteria used in our study for the diagnosis of pancreatic mucinous neoplasm. Unfortunately, we could not analyze either of the other groups with positive cytology nor the PC group given the small number of patients in each group.

## 4. Discussion

Our study demonstrated that the correlation between the morphologic ultrasonographic diagnosis of pancreatic cysts and biochemical analysis (CEA and amylase level) obtained via EUS-FNA was poor. In fact, the recommended cut-off points for CEA and amylase reported by international societies have been investigated in several studies and showed variable sensitivities and specificities. In our study, though, the cut-off point for cyst CEA level was significantly higher among patients with morphologic sonographic diagnosis of pancreatic mucinous cysts compared to another diagnosis (42.4 vs. 15.8%, chi square = 0.03, respectively). However, 57.6% of those patients had CEA <192 ng/mL, therefore, the Kappa coefficient correlation was poor. Similarly, among SCA, the correlation with CEA was poor, but was slightly better with amylase (fair agreement level). Our findings argue the role of EUS-FNA in the diagnosis of pancreatic cysts. Previous studies have addressed the utility of EUS-FNA for biochemical analysis in the diagnosis of pancreatic cystic lesions. However, the interpretation of the results is difficult given the high variability of the sensitivity and the specificity in the literature. To date, several studies have been published on the optimal cystic CEA level for the detection of mucinous cysts with values ranging from 30 to 480 ng/mL [17,18,19,20]. However, currently, the accepted and the recommended CEA value for detection of mucinous cysts is <192 ng/mL according to the ASGE guidelines [12]. A multicenter study by Brugge et al. including 120 patients have shown that CEA level higher than 192 ng/mL has a diagnostic sensitivity of 75%, a specificity of 84% and an accuracy of 79% in differentiating mucinous from non-mucinous cysts [14]. On the other hand, a very low CEA value of less than 5 ng/mL is highly suggestive of SCA or PC, as was demonstrated by a recent study showing that this cut-off value was associated with a sensitivity of 0.5 and a specificity of 0.95 for non-mucinous cysts [12]. Moreover, only 7% of patients with MCN had CEA <5 ng/ml, as compared to 100% of patients with SCA [21]. For cystic amylase level, it is an indicator of pancreatic duct communication, therefore, it is mainly used for differentiation of PC with other pancreatic cyst types. A previous pooled analysis of 12 studies including 450 patients, reported that amylase level <250 unit/L was associated with a very high specificity of 0.98 to exclude the diagnosis of PC [15]. Although the amylase cut-off point for differentiating between PC and other cysts has not been determined by the professional guidelines, however, in one study, a cut-off value for cyst amylase level of >479 units/L was associated with a sensitivity of 0.73 and a specificity of 0.9 for distinguishing PC from other cyst types [22].

Finally, more data are needed to establish the optimal cut-off values of both CEA and amylase levels, furthermore, the European Society for Gastrointestinal Endoscopy (ESGE) recommends EUS-guided FNA for cystic biochemical analysis and cytopathological examination only if it may change morphological diagnosis or patient’s management [23].

The combination of EUS morphology, cytology and CEA provided higher sensitivity, specificity and accuracy than each component test alone [15]. The diagnostic performance of cytology alone in pancreatic cyst seems to be limited, as it associated with low sensitivity and high specificity in two meta-analysis studies, 0.63 vs. 0.88 and 0.54 vs. 0.93, respectively [8,24]. In their study, Ajay et al. showed that the therapeutic decisions related to pancreatic cysts could not be based on FNA cytology results alone, as most negative and nondiagnostic specimens were associated with malignant or premalignant pathology [16]. Also, a study by Lawrence A. Shirley showed that cytologic analysis of pancreatic cyst fluid yielded no diagnostic benefit over radiologic findings alone [25]. A meta-analytical study reached the conclusion that EUS-FNA has low sensitivity but high specificity [26]. A more recent meta-analysis reached the conclusion that EUS-FNA is a reliable tool for the diagnosis of pancreatic cystic lesions; however, a more accurate algorithm is needed to reduce various biases and to improve its sensitivity in the detection of malignant cysts [27].

In the meantime, it seems that the additive role of EUS-FNA with chemical and cytological examinations is negligible in differentiating mucinous from non-mucinous pancreatic cystic lesions. The addition of molecular marker analysis in cystic fluid has been proposed to improve the diagnostic accuracy of using cytology and chemical tests alone [12]. Recently, direct optical and confocal laser endomicroscopy examination of pancreatic cysts became feasible including after intravenous administration of fluorescein in addition to cyst wall biopsy acquisition. These have also been reported to improve the diagnostic accuracy of pancreatic cysts diagnosis [25,26,27]. We could conclude that the diagnostic accuracy of EUS-FNA with chemical and cytological analysis was poor and thus could probably be waived in investigating cysts without high-risk stigmata or worrisome features by clinical and radiologic evaluation. Notably and due to its limited diagnostic role, the ASGE guidelines state that EUS-FNA is optional in asymptomatic patients in whom cross-sectional imaging demonstrates a cyst smaller than 3 cm and without a mass, lesion or dilated pancreatic duct. They recommend considering molecular testing when initial cytology and CEA results are inconclusive and when test results may alter management [12]. We still need validated tests, probably additional molecular tests, and perhaps in combination with direct optical and confocal laser endomicroscopy examinations, this may improve the diagnostic accuracy of pancreatic cysts diagnosis over the routinely available tests. 

In our study, the cytology diagnostic yield was approximately 0.35, with 100% compatibility rate with the morphological diagnosis. This finding was slightly lower than that reported in a previous study that showed a yield of cytology of approximately 45% [28].

Our study has several limitations; its retrospective design of data collection, being a single center study, operator dependency of the EUS study, and the fact that surgical histologic confirmation was lacking in most patients as they were not operated; thus, we have considered the EUS morphology as the gold standard tool for pancreatic cysts diagnosis. However, one might assume that the diagnosis of pancreatic cysts according to the EUS is doubtful, but given that all patients who had positive cytology for mucinous cysts had accurate morphological diagnosis, this reflects the accuracy of EUS morphological criteria used, and thus, can be generalized for all the study cohort.

In conclusion: EUS-FNA for cyst CEA and amylase level correlated poorly with the morphological diagnosis. The diagnosis of pancreatic cystic neoplasms should not be based solely on cyst biochemical analysis (CEA and amylase) as those tests showed poor correlation with morphological diagnosis. Our results emphasize what has been suggested by other experts in the field to obviate the need of performing EUS-FNA in patients with pancreatic cystic lesions, as the morphological appearance is highly accurate. Therefore, given the low yield of cyst fluid biochemical analysis and cytology coupled with the high accuracy of EUS morphological diagnostic accuracy, we do not recommend FNA as a complementary tool to EUS for pancreatic cystic lesion diagnosis, unless there are EUS worrisome features. Moreover, the cut-off values of CEA and amylase levels as defined by the professional societies should be revised, as our study showed lower values. Further randomized-controlled prospective studies are needed to validate our findings and to specify more accurate cut-off points for CEA and amylase levels.

## Figures and Tables

**Table 1:** Demographics, chemical and morphologic sonographic characteristics.

	Group A (IPMN)	Group B (SCA)	Group C (MCN)	Group D (PC)
Number of patients	81	14	11	5
Age (mean ± SD)	72 ± 11.1	62.6 ± 14.7	66.4 ± 11.2	56.2 ± 16.6
Gender, N (%)				
• Male	34 (42)	5 (35.7)	4 (36.4)	5 (100)
• Female	47 (58)	9 (64.3)	7 (63.6)	0
Amylase (mean ± SD)	49,288 ± 103,270	805 ± 1395	10,981 ± 28,992	19,452 ± 15,570
CEA (mean ± SD)	978 ± 2111	116 ± 399	219 ± 457	164 ± 138
Maximal cyst size (mm)	20.7 ± 12.6	31.3 ± 24.3	33.9 ± 21.9	44 ± 16.2
Cyst calcification, N (%)	3 (3.7)	2 (14.3)	1 (9.1)	0
Cyst wall thickness, N (%)	5 (6.2)	1 (7.1)	2 (18.2)	1 (20)
Cyst nodule, N (%)	5 (6.2)	0	1 (9.1)	2 (40)
Dilated Wirsung, N (%)	13 (16)	1 (7.1)	1 (9.1)	1 (20)

**Table 2:** Distribution of chemical tests among IPMN patients with agreement level.

Distribution of Chemical Analysis	CEA Level	
	>192 ng/mL	<192 ng/mL
EUS diagnosis		
Mucinous cysts (IPMN and MCN), N (%)	39 (42.4)	53 (57.6)
Another cyst type, N (%)	3 (15.8)	16 (84.2)
Sub-divided into		
• IPMN, N (%)	36 (44.4)	45 (55.6)
• Another cyst type, N (%)	6 (20)	24 (80)
• MCN, N (%)	3 (27.3)	8 (72.7)
• Another cyst type, N (%)	39 (39)	61 (61)
Agreement level		
Kappa correlation of mucinous cysts	Poor—0.130	
Kappa correlation of IPMN	Poor—0.173	

**Table 3:** Distribution of chemical tests among SCA patients with agreement level.

Distribution of Chemical Analysis	Amylase Level		CEA Level	
	<250 unit/L	>250 unit/L	<5 ng/mL	>5 ng/mL
EUS diagnosis				
• SCA, N (%)	8 (57.1)	6 (42.9)	10 (71.4)	4 (28.6)
• Another cyst type, N (%)	22 (22.7)	75 (77.3)	11 (11.3)	86 (88.7)
Agreement level				
Distribution of both or either test	Both Amylase <250 and CEA <5		Either amylase <250 or CEA <5	
	Yes		Yes	
	No		No	
EUS diagnosis				
• SCA, N (%)	7 (50)	7 (50)	11 (78.6)	3 (21.4)
• Another cyst type, N (%)	4 (4.1)	93 (95.9)	29 (29.9)	68 (70.1)
Kappa correlation of SCA	Fair—0.505		Poor—0.271	

**Table 4:** Distribution of CEA level among cytology positive mucinous cysts with agreement level.

Distribution of Chemical Analysis	CEA Level	
	>192 ng/mL	<192 ng/mL
EUS diagnosis		
• Mucinous cysts (IPMN and MCN), N (%)	13 (40.6)	19 (59.4)
• Another cyst type, N (%)	1 (14.3)	6 (85.7)
Agreement level		
Kappa correlation of mucinous cysts	Poor—0.131	

## Abbreviations

PC: pseudocysts; IPMN: Intraductal papillary mucinous neoplasm; MCN: mucinous cystic neoplasms; SCA: serous cystic adenoma; CT: computed tomography; MRI: magnetic resonance imaging; EUS: endoscopic ultrasound; FNA: fine needle aspiration; CEA: *Carcinoembryonic antigen;* ASGE: American society of gastrointestinal endoscopy; ROC: receiver operating characteristics;
